# Photoacoustic Microscopy for Multiscale Biological System Visualization and Clinical Translation

**DOI:** 10.1002/advs.202521173

**Published:** 2025-12-25

**Authors:** Tingting Wang, Jiali Chen, Liming Nie, Honghui Li

**Affiliations:** ^1^ Department of Traditional Chinese Medicine Guangdong Provincial People's Hospital (Guangdong Academy of Medical Sciences) Southern Medical University Guangzhou Guangdong China; ^2^ Medical Research Institute Guangdong Provincial People's Hospital (Guangdong Academy of Medical Sciences) Southern Medical University Guangzhou China

**Keywords:** biomedical applications, clinical translation, photoacoustic microscopy, precision medicine, technical advancements

## Abstract

Photoacoustic microscopy (PAM) has emerged as a versatile modality in biomedical research, notable for its noninvasively, high spatial resolution, and superior optical contrast. Compared with pure optical imaging techniques, PAM leverages weakly scattered ultrasonic signals for image formation, thereby achieving high‐resolution visualization of deep tissues. This review provides a comprehensive synthesis of recent advancements in PAM, encompassing technological innovations, organ‐specific applications, and emerging pathways toward clinical translation. This discussion starts by exploring the fundamental physical principles of PAM and elaborates on the enhanced performance achieved through significant advancements in high‐speed scanners, array transducers, artificial intelligence‐enhanced algorithms, and molecular agents. Pioneering applications in single‑cell analysis, hepatic microcirculation characterization, renal clearance monitoring, tumor metastasis detection, and neuroscientific discovery are surveyed to demonstrate PAM's ability. Prospective clinical uses, including intraoperative guidance and point‑of‑care diagnostics, are considered alongside persistent limitations, notably limited penetration depth and multispectral imaging speed. Future advancements are expected to hinge on multimodal integration, deeper integration of artificial intelligence, and the development of standardized protocols to accelerate clinical implementation. Ultimately, this review offers a forward‐looking perspective aimed at accelerating the translation of PAM from a laboratory tool to a clinical mainstay in the era of precision medicine.

## Graphical abstract

Photoacoustic microscopy (PAM) is a powerful biomedical imaging tool renowned for its non‐invasiveness and high resolution. This review synthesizes recent technological advances and highlights their broad applications from cellular and organ‐level to whole‐animal imaging. It further discusses the pathways and challenges for translating PAM into a clinical mainstay for precision medicine.

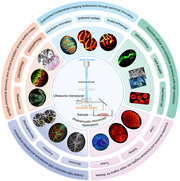

## 1 Introduction

The rapid advancement of biomedical imaging technology has constituted a fundamental driving force behind transformative progress in both basic life science research and clinical diagnostics [1]. A long‐standing paramount objective within the field has been the development of an ideal imaging modality—one capable of non‐invasive, high‐resolution, and high‐contrast interrogation of deep tissues without inflicting damage [2, 3]. However, conventional techniques have consistently fallen short of simultaneously fulfilling these criteria, each constrained by intrinsic physical limitations. Pure optical microscopy methods, such as confocal, optical coherence tomography (OCT), and two‐photon microscopy, deliver exceptional spatial resolution and molecular specificity but are severely hampered by limited penetration depth due to overwhelming optical scattering in biological tissues [4]. Conversely, clinical mainstays like ultrasound (US) and magnetic resonance imaging (MRI) provide superior penetration but often lack the spatial resolution and functional contrast necessary for visualizing cellular and subcellular structures [5].

Bridging this critical technological gap, photoacoustic imaging (PAI) has emerged as a powerful hybrid modality that synergistically combines the high contrast of optical imaging with the deep penetration of ultrasound [6]. This innovative approach is grounded in the photoacoustic effect, a physical phenomenon first documented by Alexander Graham Bell in 1880 [7]. The process is initiated when short‐pulsed laser light irradiates biological tissue, prompting the absorption of optical energy by endogenous chromophores (e.g., hemoglobin, melanin, lipids) or exogenous contrast agents [8]. This absorbed energy is converted into transient, localized thermoelastic expansion, generating broadband ultrasonic waves. By detecting these waves with conventional ultrasonic transducers and employing sophisticated reconstruction algorithms, the 3D distribution of optical absorbers can be mapped with remarkable fidelity. As a pivotal branch of PAI, photoacoustic microscopy (PAM) has established itself as a preeminent technology for high‐resolution structural, functional, and molecular imaging at the microscale [9]. Lateral resolution in PAM is primarily defined by the focusing mechanism [10]. For acoustic‐resolution PAM (AR‐PAM), resolution is constrained by the acoustic focus, which can be enhanced through a higher acoustic numerical aperture and center frequency, though this comes with trade‐offs related to depth‐of‐field and attenuation. In contrast, optical‐resolution PAM (OR‐PAM) governs lateral resolution by the optical diffraction limit, enabling tighter focusing but typically restricting imaging depth within biological tissues to approximately 1 mm. Meanwhile, axial resolution is determined by the detected acoustic bandwidth, resulting in similar axial resolution for both AR‐PAM and OR‐PAM, generally ranging from around 10 µm to greater than 1 mm, depending on the transducer bandwidth utilized [11]. Crucially, as ultrasonic scattering in tissues is approximately two to three orders of magnitude weaker than its optical counterpart, PAM achieves penetration depths of 1–3 cm for AR‐PAM and 0.5–1.2 mm for OR‐PAM in soft tissues [12]. This unique attribute allows PAM to significantly surpass the penetration limits of traditional optical microscopy while maintaining resolution superior to many clinical imaging modalities.

Notwithstanding these inherent advantages, conventional PAM systems, particularly those reliant on single‐point detection with mechanical scanning, have historically been plagued by a fundamental trade‐off between spatial resolution, imaging speed, field of view, and sampling rate [13]. This limitation has impeded their application in capturing rapid dynamic physiological processes, such as neurovascular coupling or circulating drug pharmacokinetics. To surmount these bottlenecks, the evolution of PAM technology has progressed along two primary innovation trajectories: intrinsic hardware advancements and extrinsic multimodal integration. The first pathway encompasses substantial hardware innovations, including the development of unfocused large‐area array transducers for high‐speed parallel signal acquisition, the adoption of novel scanning mechanisms (e.g., polygonal mirrors, micro‐electromechanical systems), and the implementation of advanced laser systems with versatile wavelength tuning for multispectral imaging [14]. The second pathway centers on multimodal integration, combining PAM with complementary modalities like OCT for concurrent absorption and scattering mapping, fluorescence microscopy for multiplexed molecular and hemodynamic profiling, and MRI/CT for correlative macroscopic anatomical guidance [15, 16].

Building upon these remarkable technological strides, PAM has transcended its role as a mere imaging tool to become a versatile and indispensable platform for addressing fundamental and translational biomedical challenges [17]. Its unique capacity for providing high‐resolution, label‐free insights into anatomical structures and functional dynamics has unlocked a multitude of applications across diverse physiological and pathological contexts [18, 19]. Consequently, PAM is now reshaping our understanding of complex biological systems, from deciphering neurovascular coupling and central nervous system pathologies to quantifying microcirculatory dysfunction in major organs and detecting early tumorigenic signatures [20].

**FIGURE 1:**
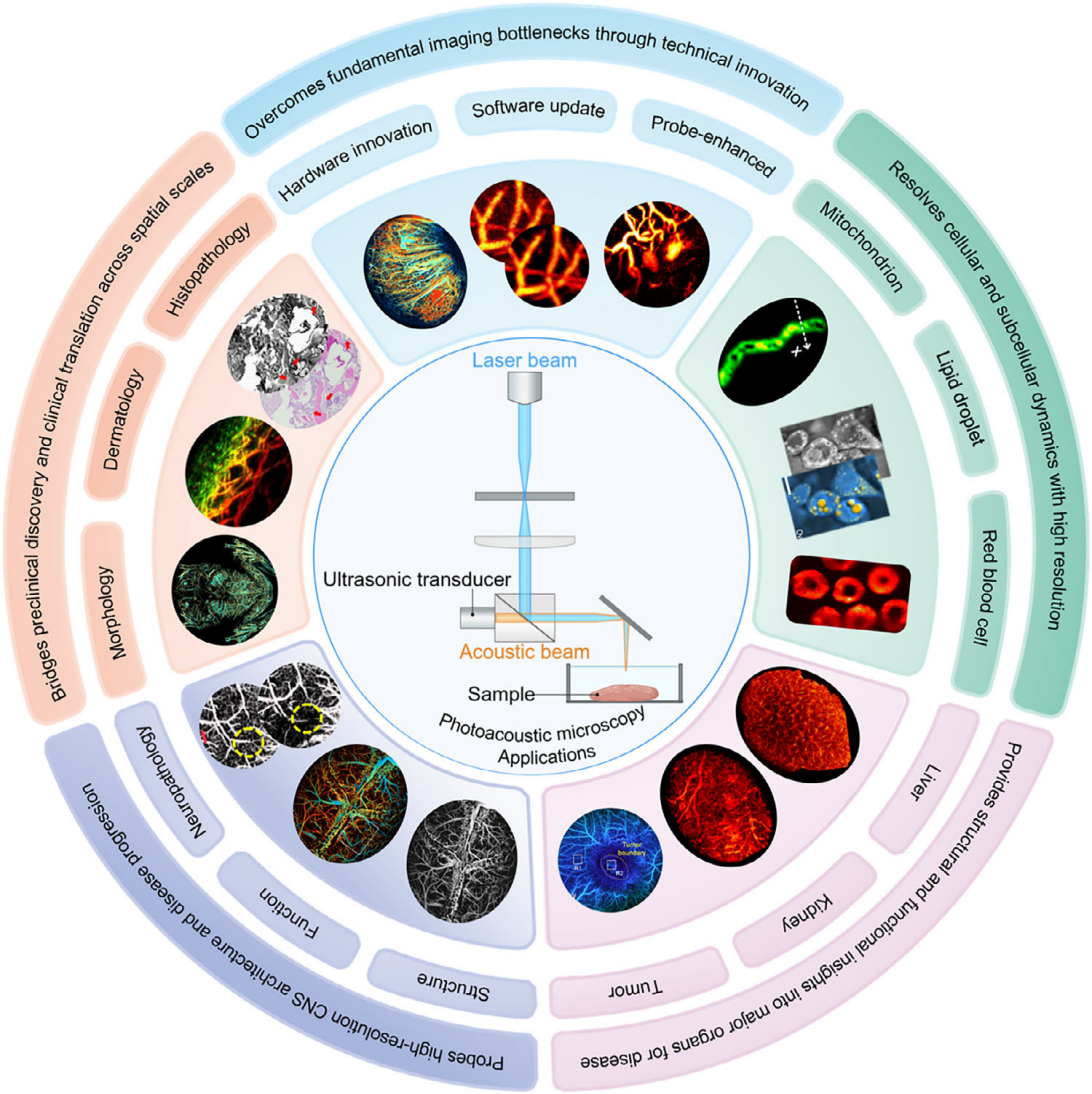
Schematic overview of key technological drivers and the broad biomedical and clinical applications of PAM. Some images were created with BioRender.com.

This comprehensive review synthesizes recent breakthroughs in PAM, highlighting its inherent multiscale capability from subcellular organelles to whole organ systems (Figure 1). Initially, we delineate the pivotal technological advancements that have addressed enduring imaging challenges, including innovations in hardware, algorithmic, and artificial intelligence (AI)‐driven enhancements, and the development of contrast agents, thereby facilitating cellular and subcellular resolution (Sections 2, 3). Building upon this foundation, we illustrate how these advancements illuminate organ‐level pathophysiology in the liver, kidneys, and tumors, while also enhancing the structural and functional understanding of the central nervous system (Sections 4, 5). Subsequently, we extend these mechanistic insights to whole‐body imaging in small animal models and explore emerging clinical applications in dermatology, oncology, and intraoperative guidance (Section 6). We also provide a systematic evaluation of the translational pathway of PAM, future directions, and ongoing challenges. By integrating microscopic mechanisms with macroscopic disease phenotypes and clinical translation within a coherent hierarchical framework, this article presents a comprehensive and forward‐looking perspective for researchers across various disciplines.

## 2 Technological Progress: Innovative Strategies for Overcoming Imaging Bottlenecks

PAM, recognized as a highly promising modality within the field of biomedical imaging, has witnessed substantial advancements in recent years through the strategic mitigation of its inherent limitations. Researchers have concentrated predominantly on innovations in hardware and enhancements driven by algorithms and artificial intelligence to address traditional constraints related to image quality, imaging speed, and functionality. These concerted efforts have resulted in comprehensive improvements in the performance of PAM, thereby facilitating its expanded application potential.

### 2.1 Hardware Innovations Enhancing PAM Performance

Hardware development plays a fundamental role in advancing PAM systems, with ongoing efforts focused on improving signal acquisition efficiency, increasing imaging speed [21], enhancing imaging resolution [22], and enabling multimodal subcellular imaging [15]. Innovations in optical–acoustic alignment are particularly critical. Traditional PAM systems utilizing off‐axis, dark‐field, or transmission‐based alignment often face a trade‐off between high resolution and deep imaging penetration, which recent advances have sought to overcome.

**FIGURE 2:**
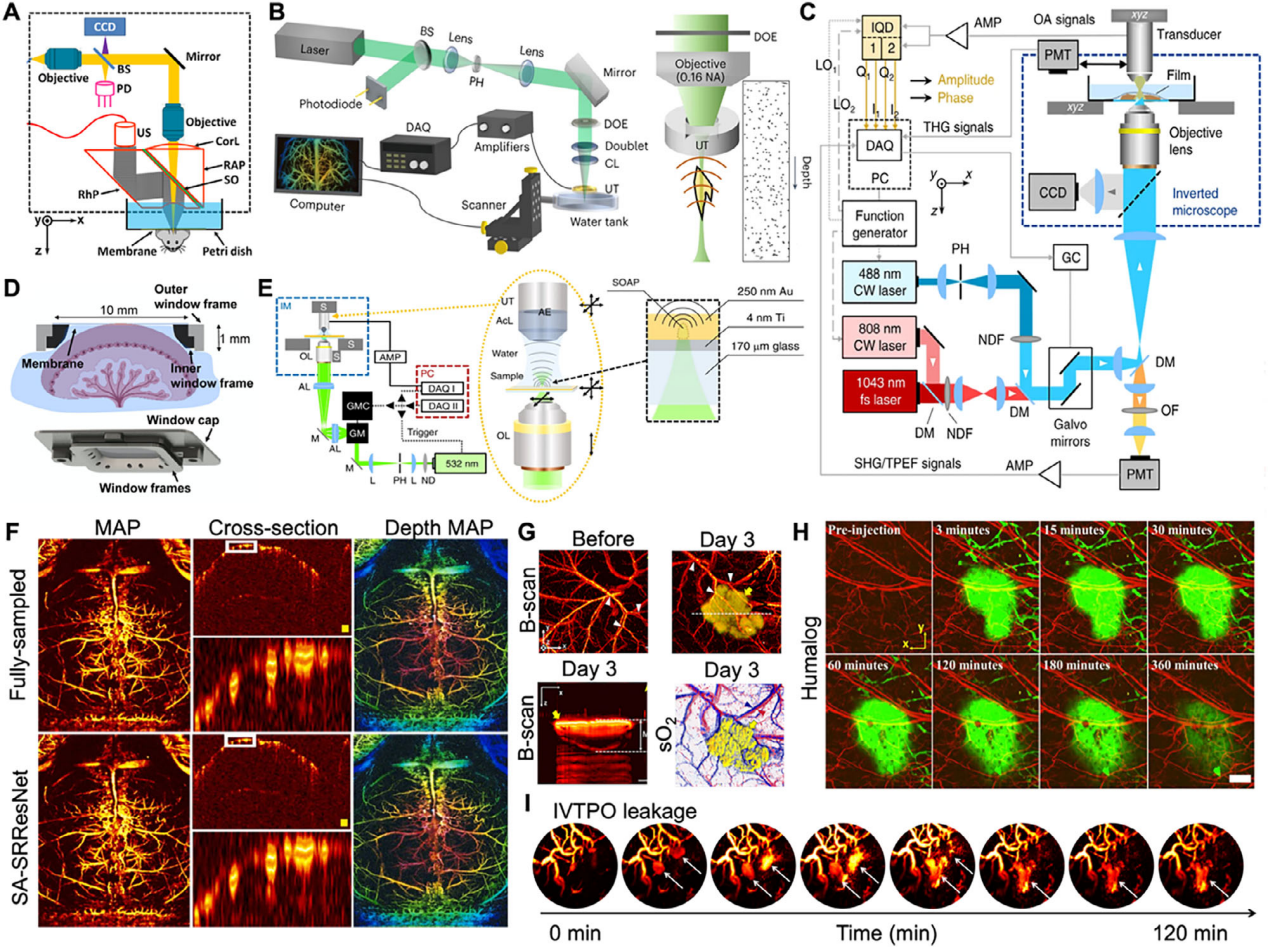
Advanced strategies for addressing imaging bottlenecks in PAM. (A) Schematic diagram illustrating the operating principle of the G2 OR‐PAM system. Reproduced with permission [23]. Copyright 2011, Optical Society of America. (B) System architecture of the needle‐beam PAM platform. Reproduced with permission [41]. Copyright 2022, Springer Nature. (C) Schematic diagram of the integrated hybrid microscopy setup. Reproduced under terms of the CC‐BY 4.0 license [45]. Copyright 2018, The Authors, published by Springer Nature. (D) Exploded view of the assembled placenta window, highlighting the structural configuration of the window frame and cap. Reproduced with permission [47]. Copyright 2024, The Authors, published by American Association for the Advancement of Science. (E) OR‐OAM system employing raster‐scanning of a 532 nm laser via galvanometric mirrors on an inverted microscope platform. Reproduced under terms of the CC‐BY 4.0 license [50]. Copyright 2020, The Authors, published by Springer Nature. (F) MAP, cross‐sectional, and depth‐encoded MAP images for data compression and SA‐SRResNet‐based processing along the *z*‐axis. Reproduced with permission [51]. Copyright 2022, The Authors, published by Elsevier GmbH. (G) In vivo multimodal PAM of subcutaneous melanomas in a murine model, demonstrating co‐registered structural and functional imaging. Reproduced under terms of the CC‐BY 4.0 license [33]. Copyright 2021, The Authors, published by the National Academy of Sciences. (H) In vivo absorption profiles of sulfo‐Cy7.5‐labeled lispro within Humalog formulations, acquired in a mouse ear model. Reproduced under terms of the CC‐BY 4.0 license [85]. Copyright 2022, The Authors, published by Elsevier GmbH. (I) Serial PAM images of cerebral vasculature in a GBM‐bearing mouse at advanced disease stage, before and after intravenous injection of IVTPO at the indicated time points. Reproduced with permission [86]. Copyright 2025, The Authors, published by American Association for the Advancement of Science.

Reflective opto‐ultrasound combiners (OUCs) enable seamless high‐resolution imaging via probe‐arm scanning, though their constrained numerical aperture (NA) can limit sensitivity [23], as shown in Figure 2A. Since their introduction in 2008, OUCs have significantly improved beam alignment, achieving tightly focused illumination with lateral resolutions below 5 µm [11]. This configuration ensures precise coaxial alignment, greatly enhancing signal collection efficiency and system sensitivity [24], and has been successfully applied in clinical settings such as dynamic imaging of human finger vasculature [25, 26]. A key limitation of conventional OUCs is their inability to support high optical NA, leading to acoustic energy loss from impedance mismatch [27]. To address sensitivity issues without sacrificing alignment, Ring‐shaped ultrasound transducers (RUTs) offer an alternative by permitting coaxial alignment without sacrificing sensitivity [28]. Their annular design allows central light transmission, improving signal detail and image quality, which has proven valuable for pathological examination of bone and other tissues [29]. However, RUTs also suffer from acoustic energy loss due to impedance mismatch, restricting their potential for resolution enhancement [30]. To address this, transparent ultrasound transducers (TUTs) fabricated from optically clear materials were developed. Introduced in 2019, TUTs maintain high acoustic sensitivity while enabling integration with high‐NA objective lenses [31]. This transparency supports sophisticated multimodal system designs, enabling the seamless integration of PAI, US, OCT, and fluorescence modalities in a quadruple fusion imaging system [32, 33]. Furthermore, TUTs are proving instrumental in developing compact opto‐ultrasound biosensors for wearable and mobile devices, facilitating the simultaneous acquisition of photoplethysmography and ultrasound signals for vital sign monitoring [34]. To broaden TUT applicability, recent advancements have also focused on optimizing TUT bandwidth, with designs employing polymethyl methacrylate as a matching layer achieving significantly broader bandwidths and improved axial resolution in PAM [35]. Building upon these innovations, semi‐transparent ultrasound transducers have been developed to facilitate simplified, handheld multimodal imaging, enabling simultaneous photoacoustic and laser‐induced ultrasound imaging via a single laser shot, thereby eliminating the need for an external US pulser and enhancing system compactness for preclinical and clinical studies [36].

In scanning technology, micro‐electro‐mechanical system (MEMS) scanners provide a compact and cost‐effective alternative to conventional mechanical stages, particularly for OR‐PAM in the visible spectrum [37]. Galvanometer scanners offer high stability and precise vector scanning, making them well‐suited for vascular imaging [38, 39]. For even higher speeds, polygonal mirror scanners enable large‐field applications such as whole‐brain imaging [40]. To overcome the shallow depth‐of‐field limitation in OR‐PAM, needle‐shaped beam PAM has been introduced [41]. This technique employs customized diffractive optical elements to generate a beam with a consistent diameter and uniform intensity over an extended focal region, increasing the depth of field by approximately 28‐fold (Figure 2B). Similarly, for UV‐PAM, specialized UV metalenses and metasurface‐assisted strategies have been developed to significantly extend the depth of focus (e.g., up to 290 µm) while preserving high lateral resolution, crucially enabling fast, label‐free histology imaging of unprocessed tissues with uneven surfaces for pathological diagnosis [42, 43]. This advancement permits high‐resolution imaging of uneven surfaces and high‐quality volumetric data acquisition without z‐scanning, promising for slide‐free histology and in vivo organ microscopy. Regarding light sources and system architecture, PAM has conventionally relied on pulsed lasers for signal excitation [44]. A significant paradigm shift from this paradigm is frequency‐domain optoacoustic microscopy, which uses intensity‐modulated continuous‐wave laser diodes instead of high‐energy pulses [45]. This innovation circumvents the limitations of complex pulsed lasers, allowing for high repetition rates and simultaneous multi‐wavelength illumination for spectral imaging (Figure 2C). Ongoing system development also includes dual‐mode platforms integrating both OR‐PAM and AR‐PAM. Furthermore, there is a growing emphasis on portability and miniaturization, with implantable systems incorporating integrated sensors and actuators currently under development [46]. Respiratory motion artifacts and limited penetration depth pose major challenges for deep‐tissue PAM imaging. To address this, Zhu et al. created an implantable placental window using a biocompatible titanium alloy (Figure 2D), integrated with ultrafast functional PAM (UFF‐PAM) [47]. This platform enables long‐term, non‐invasive observation of placental microvasculature and molecular activity. By securing the window's outer frame with a 3D‐printed clamp, respiratory motion effects are significantly reduced. Integrated with UFF‐PAM, it provides high‐resolution monitoring of placental hemodynamics, aiding in the study of placental function and adverse pregnancy outcomes.

### 2.2 Algorithmic and AI‐Driven Enhancements in Image Quality

Beyond hardware, algorithmic and AI techniques are increasingly pivotal for enhancing PAM image quality, acquisition speed, and functional capacity [48]. Conventional reconstruction algorithms, such as synthetic aperture focusing and deconvolution, effectively suppress noise and artifacts, improving resolution and clarity [49]. A key strategy involves correcting the system's point spread function (PSF). In OR‐PAM, this is complicated by spatially‐dependent signal distortions and the time‐resolved nature of optoacoustic signals, encapsulated in the 4D total impulse response (TIR). Recent advancements using spatially‐distributed optoacoustic point sources have enabled spatially‐dependent TIR correction (Figure 2E), boosting the signal‐to‐noise ratio by over 10 dB and axial resolution by ∼30% [50]. The rise of deep learning (DL) has catalyzed the development of data‐driven methods for PAM image processing as shown in Figure 2F [51]. These include image restoration techniques where DL models recover high‐quality images from sparse or corrupted data, enhancing detail and suppressing noise [52]. For instance, a unified network (UPAMNet) has been developed that leverages deep knowledge priors, including spatial, oriented, and positional attention, to simultaneously address PAM image super‐resolution and denoising, significantly improving image quality and acquisition efficiency across diverse datasets [53]. Specific neural networks are trained to distinguish true signals from noise, improving signal fidelity [54]. AI‐driven approaches also compensate for geometric distortions and artifacts, while DL‐based super‐resolution techniques reconstruct high‐resolution images from lower‐resolution acquisitions, revealing fine anatomical structures [55, 56]. AI is being deployed to tackle the fundamental challenge of tissue‐induced optical aberrations in OR‐PAM. The DeepCAO framework exemplifies this, using a deep learning network trained on both simulated and experimental data to computationally correct aberrations without any hardware modifications [57]. Furthermore, algorithms play a crucial role in adapting to complex in vivo environments. For instance, an automatic skin profile detection algorithm analyzes signal amplitudes in volumetric PAM data to precisely map the skin surface [58]. This is complemented by an auto‐fit scanning mechanism that dynamically adjusts the ultrasonic focus to effectively track subcutaneous vessel layers, thereby significantly enhancing image quality on contoured surfaces. To address the variability in image degradation associated with AR‐PAM, which is impacted by imaging depth and transducer frequency, a hybrid approach integrates a deep convolutional neural network as a “plug‐and‐play” prior within a model‐based optimization framework [59]. This approach adaptively manages different distortions, learns vasculature statistics, and is guided by the physical PSF, resulting in superior signal‐to‐noise ratio, contrast‐to‐noise ratio, and image fidelity in vivo. Looking ahead, the role of AI is expanding beyond mere image enhancement to address more complex analytical challenges. Deep learning models are increasingly being utilized to enhance the accuracy of quantitative hemodynamic mapping, such as the quantification of blood oxygen saturation (sO_2_), by reducing wavelength‐dependent artifacts and scattering effects. Additionally, DL‐based methodologies are facilitating more robust and rapid spectral unmixing of overlapping chromophore signals in low‐signal environments. Perhaps most promisingly, AI is advancing automated diagnostic classification, wherein PAM images can be directly input into trained networks to identify pathological states, such as tumor margins and disease grading [60]. This advancement is exemplified in preliminary clinical studies discussed in Section 6.2, thereby propelling the field toward intelligent, operator‐independent analysis. These advancements underscore the transformative impact of AI on PAM, enabling more detailed and reliable biological imaging.

### 2.3 Contrast Agent Technology Enhancing PAM Applications

The continuous development of contrast agents has substantially broadened PAM's application scope, facilitating its evolution from structural imaging toward functional and molecular profiling [61, 62]. PAM agents are categorized into endogenous and exogenous types, each providing distinct contrast mechanisms that collectively enhance the system's versatility [63].

Endogenous agents leverage naturally occurring chromophores. Hemoglobin is the most widely used; its oxygenated and deoxygenated forms exhibit characteristic absorption peaks in the visible spectrum, enabling PAM to non‐invasively quantify sO_2_ and monitor tissue metabolism [64]. Melanin, with strong absorption in the first near‐infrared window (NIR‐I, 700–900 nm), allows precise detection and demarcation of melanoma lesions [65]. This capability is compellingly demonstrated in vivo, where multispectral PAM utilizes melanin as an intrinsic contrast agent to effectively identify subcutaneous melanoma [33]. This enables precise visualization of the tumor, its surrounding vasculature, and it facilitates accurate measurement of its thickness without the need for exogenous probes (Figure 2G). Lipids display specific absorption in the second near‐infrared window (NIR‐II, e.g., 1210 and 1720 nm), supporting label‐free visualization of pathological conditions like fatty liver disease and atherosclerosis [66, 67]. Additionally, water absorption characteristics at wavelengths such as 970 and 1190 nm provide a means to evaluate tissue edema [68]. These endogenous agents supply rich physiological information without external administration, underscoring a key advantage for clinical translation [69]. Exogenous agents augment PAM performance by improving the signal‐to‐noise ratio, penetration depth, and molecular specificity [70]. Clinically approved small‐molecule dyes like indocyanine green (ICG) and methylene blue (MB) are commonly employed. ICG, with strong absorption around 800–850 nm, is suitable for vascular imaging [71], liver function studies [72], and lymphatic mapping [73]. Building upon ICG's clinical utility, size‐tunable ICG J‐aggregates have been developed as a versatile contrast agent platform, enabling direct functionalization with targeting moieties and demonstrating enhanced in vivo visualization and improved contrast‐to‐noise ratios in targeted near‐infrared PAM [74]. Nanomaterial‐based agents, including gold nanorods with tunable NIR‐II absorption and polypyrene nanoparticles, can be functionalized with targeting ligands for specific biomarker imaging [75, 76]. Expanding this category, novel ultrasmall biocompatible bornite (Cu5FeS4) nanocrystals have emerged as promising NIR‐II contrast agents, demonstrating superior photoacoustic signal amplitude compared to traditional agents like ICG and gold nanorods, thereby enabling enhanced deep tissue imaging [77]. More recently, genetically encoded agents such as BphP1 [78] and IRFP720 [79] have enabled long‐term, dynamic molecular imaging of specific cellular processes, offering genetically programmable tools for biological research [80].

Crucially, multispectral imaging techniques are essential for differentiating and quantifying multiple agents simultaneously [81]. By acquiring data at several wavelengths and applying spectral unmixing algorithms, PAM can resolve the spatial distribution and concentration of various absorbers, allowing concurrent observation of diverse physiological parameters [82]. These advances have considerably expanded PAM's utility: in oncology, targeted agents visualize tumor‐specific markers [83]. In neuroscience, genetically encoded calcium indicators enable large‐scale imaging of neuronal activity [84]. PAM has demonstrated significant utility in pharmaceutical research, as illustrated by a study employing near‐infrared dye‐labeled insulin lispro to visualize and model its subcutaneous absorption dynamics [85]. The findings indicate that diffusion and vascular permeation, rather than hexamer dissociation, primarily determine the absorption rate (Figure 2H). This study highlights PAM's distinctive ability to elucidate the in vivo behavior of protein therapeutics and inform the rational design of drug formulations. The ongoing advancement in rational molecular design is enhancing targeted imaging in complex disease models. A key example is the amphiphilic hemicyanine dye IVTPO [86], engineered with an electron donor‐π‐acceptor system to penetrate the blood–brain barrier (BBB). This dye allows high‐resolution, dual‐modal fluorescence/PAI of glioblastoma through the skull and can detect early tumor‐related vascular leakage before significant BBB disruption, outperforming traditional dyes in targeting and imaging efficacy (Figure 2I).

In summary, through the provision of specific contrast mechanisms, signal enhancement, and molecular targeting capabilities, contrast agent technology has profoundly extended the functional reach of PAM. From label‐free endogenous imaging to precision molecular sensing, these developments are solidifying PAM's role as a versatile platform for both fundamental biomedical research and clinical applications [87].

## 3 PAM Applications in Single‐Cell and Subcellular Imaging

**FIGURE 3:**
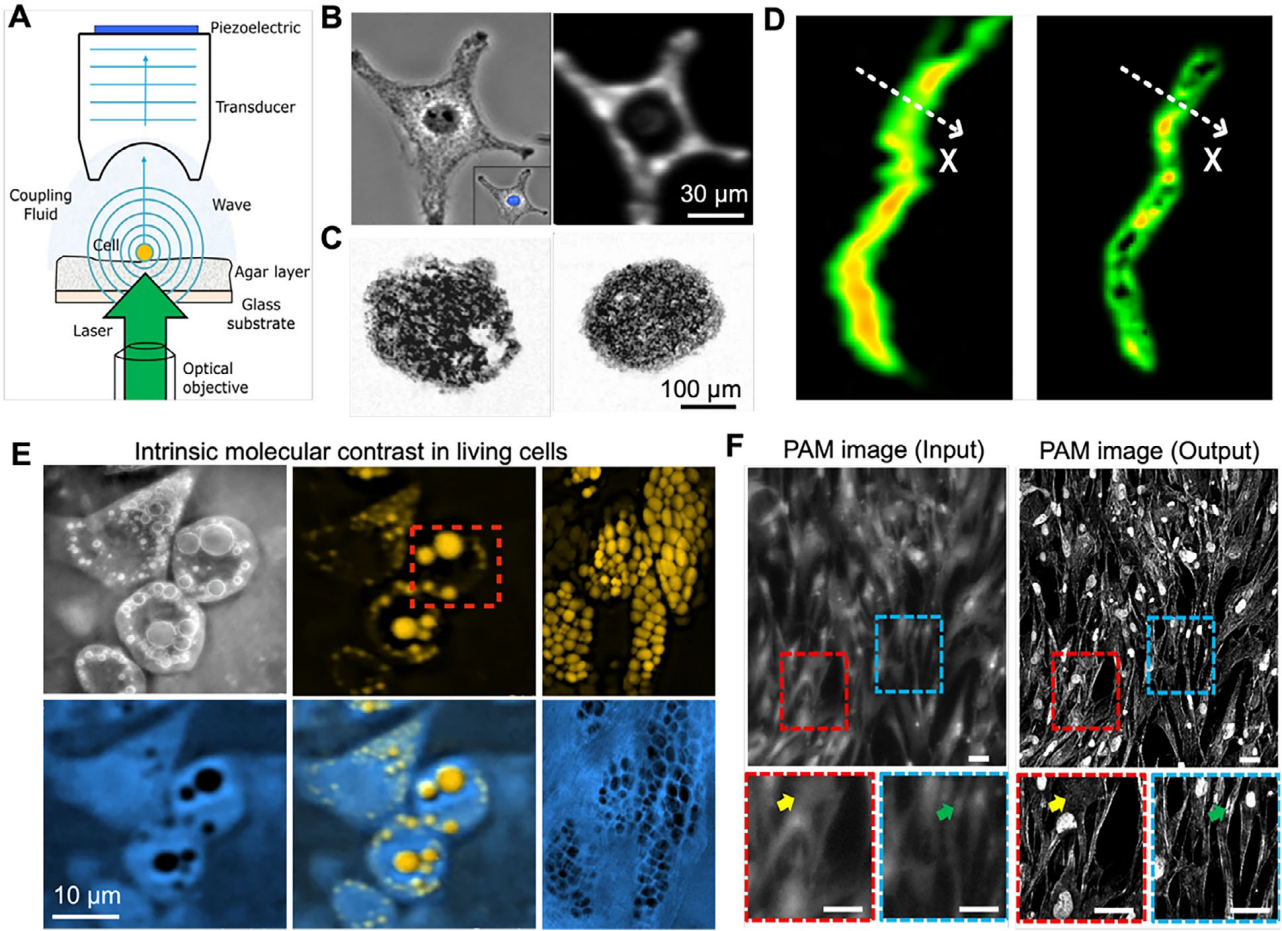
High‐resolution PAM for single‐cell and subcellular imaging. (A) Schematic illustrating photoacoustic signal generation and propagation from an individual cell. Reproduced with permission [89]. Copyright 2015, International Society for Advancement of Cytometry. (B) Bright‐field optical image and corresponding photoacoustic image of a single formalin‐fixed B16‐F1 melanoma cell. Reproduced with permission [90]. Copyright 2013, The Authors, published by Elsevier GmbH. (C) Photoacoustic images of an embryoid body and a multicellular tumor spheroid following MTT staining. Reproduced with permission [92]. Copyright 2011, WILEY‐VCH Verlag GmbH & Co. KGaA, Weinheim. (D) Mitochondrial morphology visualized by conventional PAM and photoacoustic nanoscopy, demonstrating the superior resolution for subcellular structures. Reproduced under terms of the CC‐BY 4.0 license [93]. Copyright 2014, The Authors, published by SPIE. (E) Bright‐field visible‐light image and MiROM micrographs of differentiated 3T3‐L1 cells. Reproduced with permission [100]. Copyright 2020, Springer Nature. (F) Representative comparison of cross‐domain images generated by the IREN framework. Scale bars: 50 µm. Reproduced under terms of the CC‐BY 4.0 license [103]. Copyright 2024, The Authors, published by Springer Nature.

The development of compact and high‐performance PAM systems has significantly advanced cellular imaging capabilities. PAM has established itself as a transformative tool for investigating biological systems at cellular and subcellular levels, providing unique insights into cell morphology, organelle distribution, and intracellular metabolic activities [88]. As illustrated in Figure 3A, the fundamental principle of PAM involves irradiating single cells with a pulsed laser; the subsequent absorption of light generates ultrasonic waves via the photoacoustic effect, which are then detected by a high‐frequency transducer to reconstruct high‐resolution cellular images [89]. By leveraging both endogenous optical absorption contrasts and exogenous probes, PAM enables non‐invasive, high‐resolution visualization of cellular structures and dynamic processes while overcoming limitations inherent to traditional fluorescence microscopy, including photobleaching and phototoxicity. The technology's versatility allows it to address a broad spectrum of biological questions, from fundamental cell biology to clinical diagnostics, through its unique combination of optical contrast and ultrasonic resolution.

### 3.1 High‐Resolution Cellular and Subcellular Imaging

The development of compact and high‐performance PAM systems has significantly advanced cellular imaging capabilities. For example, in the area of single‐cell imaging of melanoma cells (Figure 3B), the utilization of ultrasound transducers with elevated central frequencies has been demonstrated to markedly improve lateral resolution, thereby facilitating a detailed visualization of intracellular melanin distribution [90]. The robust correlation observed between the photoacoustic signal and the optically measured melanin density substantiates the efficacy of PAM in achieving label‐free subcellular mapping. A compact fiber laser‐based PAM system measuring 45 × 56 × 13 cm^3^ with a 50‐kHz pulse repetition rate enables fast scanning and high sensitivity at 1064 nm wavelength. This system provides sufficient sensitivity to image small blood vessels while offering excellent optical absorption contrast between melanin and hemoglobin, allowing label‐free in vitro imaging of melanoma cells in flowing bovine blood with simultaneous measurement of cell size and flow dynamics [91]. While PAM provides noninvasive tissue examination with high resolution and penetration depth using nonionizing waves, many cell types lack intrinsic pigments for detection. To address this limitation, researchers have introduced 1‐(4,5‐ dimethyl‐2‐thiazolyl)‐3,5‐diphenylformazan (MTT formazan) as a stable and nontoxic contrast agent for high‐resolution PAM imaging of various cell types. This approach enables the noninvasive spatial resolution of individual cells and cell clusters (Figure 3C), allowing visualization of cell distribution, proliferation, and invasion profiles within 3D porous scaffolds [92].

PAM achieves exceptional spatial resolution through multiple advanced technical approaches that have been developed to address specific imaging challenges. UV‐PAM capitalizes on the strong absorption of nucleic acids in the UV spectrum to visualize unlabeled cell nuclei with remarkable clarity [28]. This technique has been successfully applied to various biological systems, with one notable study demonstrating UV‐PAM imaging of cell nuclei in mouse intestinal tissue with 700 nm resolution using a 266 nm laser source coupled with a ring‐shaped ultrasonic transducer. Beyond UV‐PAM, super‐resolution techniques have pushed the boundaries of cellular imaging beyond the optical diffraction limit. Nonlinear photoacoustic methods have achieved resolutions as fine as 88 nm, sufficient for detailed mitochondrial imaging (Figure 3D) [93]. A highly effective strategy for nanoscale imaging at the subcellular level involves the integration of expansion microscopy (ExM) with PAM. This technique, known as expansion fluorescence and PAM (ExFLPAM), utilizes physical sample enlargement in conjunction with on‐demand chromogenic labeling to transform fluorescent protein signals into absorption contrasts. This combination facilitates high‐resolution volumetric PAM of expanded cells and tissues [94]. Additionally, the development of axially multifocal metalenses, which employ sub‐micrometer‐thick nanopillars, has resulted in the ability to generate elongated beams with uniform intensity and an extended depth of field reaching up to 500 µm. This innovation is critical for enabling 3D volumetric photoacoustic imaging of neuromelanin in complex live brain organoids, thereby advancing research in neurodegenerative diseases [95]. Complementing these approaches, the development of TUTs has further advanced this field by enabling improved numerical aperture and sensitivity, which is beneficial for diverse cancer imaging applications, including clear visualization of cancer cell nuclei in brain tissue [96, 97], and comprehensive multimodal tumor analysis [98]. These innovations in transducer technology have collectively enhanced our ability to study nuclear morphology and organization without the need for staining or labeling procedures, opening new possibilities for non‐invasive cellular analysis.

### 3.2 Mid‐Infrared PAM for Biochemical Imaging

Recent advances in mid‐infrared PAM (MIR‐PAM) have significantly expanded the biochemical imaging capabilities at the cellular level, providing unprecedented access to molecular information without the need for labels. A novel methodology has been developed to address the intrinsic limitations of traditional MIR microscopy, specifically the constraints of diffraction‐limited resolution and the pronounced water background interference. This approach utilizes the Grüneisen relaxation effect combined with a pulsed UV laser to enable highly localized photoacoustic detection [99]. Consequently, this technique attains UV‐level resolution for MIR imaging of lipids and proteins, surpassing the optical diffraction limit by at least an order of magnitude, while simultaneously mitigating the interference from the water background. MIR‐PAM extends cellular imaging capabilities by targeting specific molecular vibration modes, allowing visualization of lipid and protein distributions within single cells. Researchers have achieved a remarkable 0.26 µm resolution by combining UV localization with MIR detection (Figure 3E), enabling detailed mapping of subcellular components [100]. This high‐resolution chemical imaging has proven particularly valuable for studying cellular metabolism and composition changes in response to environmental stimuli or pathological conditions. Further enhancing these capabilities, the development of the depth‐gated mid‐infrared optoacoustic sensor enables non‐invasive glucose detection in blood‐rich skin volumes through depth‐selective molecular localization, offering new avenues for studying cellular metabolism [101]. This technology represents a significant advancement in metabolic monitoring at the cellular level, providing insights into nutrient uptake and utilization processes. Additionally, mid‐infrared optoacoustic microscopy (MiROM) provides label‐free cellular imaging in thick tissues, overcoming water absorption limitations of conventional infrared methods and supporting cellular‐level histopathological assessments without extensive sample processing [102]. To address the spatial resolution limitations of mid‐infrared PAM, an explainable deep learning framework has been developed that transforms low‐resolution MIR‐PAM images into high‐resolution, virtual fluorescence‐stained images. This unsupervised approach enables label‐free, high‐resolution identification of subcellular structures such as nuclei and filamentous actin in human cardiac fibroblasts (Figure 3F), matching the quality of confocal fluorescence microscopy while preserving the biochemical specificity of MIR‐PAM [103]. The integration of artificial intelligence with MIR‐PAM has substantially expanded the applicability of this technology for detailed cellular analysis, particularly for studies where traditional staining methods may interfere with cellular function or viability.

### 3.3 Functional and Metabolic Imaging Applications

Beyond structural characterization, PAM provides valuable functional and metabolic information at the cellular level, enabling researchers to connect morphological observations with functional outcomes. The technique can monitor cellular oxygen metabolism by measuring hemoglobin oxygen saturation and blood flow in surrounding microvessels [104]. This capability has been instrumental in studying cellular responses to hypoxic conditions and understanding metabolic adaptations in various pathological states. The development of high‐sensitivity multi‐spectral OR‐PAM systems has further enhanced these functional imaging capabilities by enabling simultaneous acquisition of structural and metabolic information across multiple wavelengths. Expanding these functional applications, multiphoton PAM has enabled label‐free imaging of metabolic cofactors such as NAD(P)H, a key indicator of cellular energy metabolism. This approach achieves significant imaging depths in complex systems like brain organoids, surpassing conventional fluorescence microscopy limitations [105]. The ability to detect NAD(P)H without labels provides a powerful tool for monitoring metabolic changes associated with neuronal activity, stem cell differentiation, and cellular stress responses. Such deep‐tissue metabolic imaging creates new possibilities for studying cellular functions in 3D microenvironments that more closely resemble in vivo conditions.

For longitudinal cellular tracking, integrated PAM‐OCT imaging platforms enable monitoring of transplanted stem cells in living eyes using chainlike gold nanoparticle labels, providing precise anatomical localization over extended periods and offering valuable insights into cell migration and therapy efficacy in regenerative medicine [106, 107]. This combined approach leverages the strengths of both imaging modalities to provide comprehensive information about cell location, viability, and function in living systems. The integration of PAM with other imaging technologies has emerged as a powerful strategy for addressing complex biological questions that require multiple types of information. Furthermore, the integration of PAM with microfluidic technologies has facilitated high‐throughput single‐cell analysis. A notable development is the self‐calibrated photoacoustic flow cytometry system, which utilizes a 360° panoramic transducer array for parallel detection of multiple cells, enabling rapid screening of cellular properties [108]. These high‐throughput approaches are particularly valuable for cancer research, immunology, and drug discovery, where large‐scale single‐cell analysis is essential for understanding population heterogeneity and identifying rare cell subtypes. The combination of PAM with microsystems has opened new avenues for automated cell analysis with minimal operator intervention.

Future developments in PAM technology for cellular imaging will likely focus on improving imaging speed, resolution, and molecular specificity, addressing current limitations to expand application possibilities. The integration of artificial intelligence for image analysis and interpretation, coupled with the development of novel contrast agents targeting specific organelles, will further enhance PAM's utility in cell biology research. Additionally, combining PAM with complementary modalities such as adaptive optics and Raman microscopy may provide more comprehensive cellular characterization, ultimately advancing our understanding of fundamental biological processes in health and disease. As these technological advancements continue to evolve, PAM is poised to become an increasingly indispensable tool for single‐cell analysis across diverse biological and biomedical research applications.

## 4 PAM Provides Structural and Functional Insights Into Major Organs and Tumors

### 4.1 Multiscale PAM for Investigating Hepatic Pathologies

**FIGURE 4:**
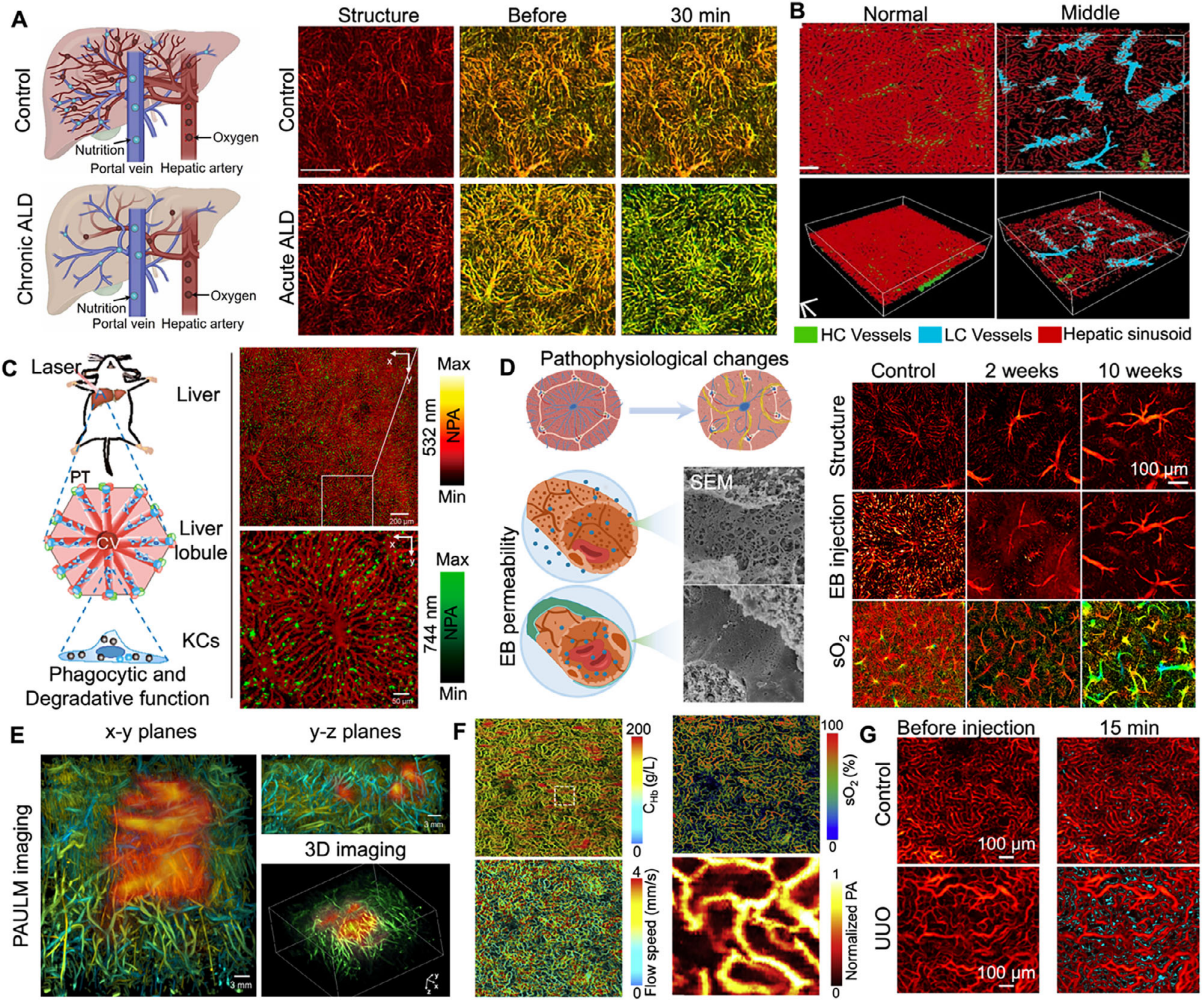
Multiscale PAM for investigating hepatic and renal pathologies. (A) Longitudinal monitoring of LFR in murine hepatic subregions, accompanied by microstructural and oxygen metabolism imaging in an acute ALD model. Reproduced with permission [110]. Copyright 2023, The Authors, published by Elsevier GmbH. (B) Quantitative assessment of vascular coverage in mouse liver tissues under physiological conditions and at distinct stages of disease progression. Reproduced under terms of the CC‐BY license [112]. Copyright 2025, The Authors, published by Elsevier GmbH. (C) In vivo photoacoustic visualization of Kupffer cells distribution within the hepatic lobular architecture of mice. Reproduced with permission [113]. Copyright 2019, American Chemical Society. (D) Representative PAM images of liver lobules following EB injection in control mice and in murine models at progressive stages of liver fibrosis. Reproduced with permission [114]. Copyright 2025, Radiological Society of North America. (E) 3D‐PAULM of AAV‐Cre‐induced BphP1 expression in the liver. Reproduced with permission [116]. Copyright 2025, The Authors, published by Springer Nature. (F) Quantitative measurements of the C_Hb_, sO_2_, blood flow speed, and a close‐up view of the white boxed region in the kidney microvasculature. Reproduced with permission [120]. Copyright 2021, Elsevier. (G) Time‐lapse PAM series documenting dynamic Evans Blue extravasation in the kidney. Reproduced with permission [125]. Copyright 2025, The Authors, published by Chinese Optics Letters.

PAM has emerged as a powerful tool for studying liver diseases, providing high‐resolution insights into hepatic microvascular structure [109], tissue oxygenation [110], and molecular events [111]. A key application lies in the quantitative assessment of liver function. Through an empirical mathematical model derived from photoacoustic signals, liver function reserve (LFR) parameters can be quantified, demonstrating prolonged ICG half‐life in alcoholic liver disease (ALD) models. This scalable PAM approach shows considerable promise for noninvasive LFR assessment in early diagnosis and management of alcohol‐related liver diseases as shown in Figure 4A [110]. Beyond global functional assessment, PAM excels at quantifying subtle microvascular changes. In non‐alcoholic steatohepatitis (NASH), researchers have combined OR‐PAM with a modular liver window and 3D‐printed imaging (Figure 4B) stent to facilitate longitudinal studies, providing unprecedented insight into the microcirculatory failure cascade [112]. Expanding into molecular and immunological imaging, PAM enables precise diagnosis of specific liver conditions. In liver immunology, a pioneering nano pomegranate probe enables dual‐modal fluorescence photoacoustic imaging of Kupffer cells (KCs) as shown in Figure 4C, revealing their strategic spatial distribution and functional heterogeneity along the central vein‐to‐portal vein axis within the liver lobule [113]. PAM's capability for non‐invasive monitoring is also particularly promising in the context of liver fibrosis. In a carbon tetrachloride‐induced mouse model, PAM effectively tracked dynamic changes within hepatic sinusoids and revealed significant correlations between quantitative PAM parameters and fibrosis stages: increased vascular lacunarity corresponded with collagen deposition, decreased blood oxygen saturation aligned with upregulation of hypoxia‐inducible factor 1α, and altered Evans blue permeability reflected sinusoidal capillarization (Figure 4D). These findings underscore PAM's value in monitoring microvascular alterations during liver fibrosis progression [114]. Addressing technical challenges in liver imaging, such as respiratory motion artifacts and limited probe performance, innovative solutions have been developed. A drawer‐style abdominal window model significantly reduces motion interference, enabling long‐term, high‐quality fluorescence and photoacoustic imaging of the liver for over 10 days [115]. Furthermore, the Blvra^−^/^−^ gene knockout mouse model enhances the performance of near‐infrared optical probes such as BphP1 in deep tissues (Figure 4E), significantly improving the signal‐to‐noise ratio in organs including the liver [116]. This genetic approach substantially boosts the sensitivity of molecular PAM imaging for hepatic applications. For molecular‐specific diagnoses, such as in drug‐induced liver injury (DILI)—a considerable diagnostic challenge—a novel bienzyme‐locked activatable photoacoustic probe (SH‐NAL) has been developed. This probe is selectively activated by two DILI‐associated enzymes (LAP and APN), allowing differentiation of DILI from other liver conditions such as hepatitis and cirrhosis with a high signal‐to‐noise ratio in both in vivo and serum applications [117]. Similarly, in hepatocellular carcinoma, high‐resolution PAM has characterized disorganized tumor vasculature, while in ALD models, dual‐wavelength spectral unmixing has revealed significant decreases in hepatic oxygen metabolism alongside abnormal lobular restructuring.

In summary, PAM provides a versatile platform for liver disease research, enabling quantitative assessment of microvascular pathology, specific molecular imaging for precise diagnosis, and longitudinal monitoring of disease progression and treatment response [118]. Future developments involving more sensitive molecular probes and faster, integrated imaging systems will further solidify PAM's role in advancing hepatological research and clinical practice.

### 4.2 Multiscale PAM for Investigating Renal Pathologies

PAM has emerged as a powerful tool for investigating kidney diseases, providing high‐resolution insights into renal microvascular architecture, functional dynamics, and molecular pathology. Its label‐free functional imaging capability is particularly valuable for studying pathological processes including fibrosis, acute kidney injury (AKI), and drug‐induced nephrotoxicity. PAM effectively quantifies functional and structural alterations in renal tissue across various disease contexts. In hereditary tyrosinemia type I (HT1) models, it has detected severe tubular structural damage and significantly reduced renal blood oxygen saturation, highlighting its potential for in vivo evaluation of metabolic disease‐associated renal lesions [119]. For acute kidney injury, a label‐free PAM technique enables simultaneous quantification of the total concentration of hemoglobin (C_Hb_), sO_2_, blood flow speed in peritubular capillaries (Figure 4F), allowing assessment of regional oxygen metabolism. In sepsis‐induced AKI models, this approach revealed significant decreases in capillary oxygen saturation and renal ATP levels, despite minimal changes in capillary flow and plasma creatinine, demonstrating PAM's ability to provide integrated hemodynamic and metabolic insights into AKI pathophysiology [120]. In the domain of molecular imaging, PAM has proven instrumental in visualizing pathogenic immune complex deposition. One significant study employed PAM to track immunoglobulin A (IgA) deposition in mouse glomeruli with approximately three‐micron resolution, enabling detailed observation of galactose‐deficient IgA persistence in kidney tissues and providing direct visual evidence of its link to renal injury [121]. For chronic kidney disease (CKD) assessment, high‐resolution raster scanning optoacoustic mesoscopy (RSOM) achieves label‐free imaging of renal dimensions and vascular networks, clearly delineating intrarenal vascular structures and differentiating oxygenated from deoxygenated regions. In α8 integrin‐knockout CKD models, PAM revealed reduced kidney size and vascular area compared to wild‐type controls, indicating disease‐associated vascular rarefaction [122]. Furthermore, PAM enables direct quantification of renal fibrosis through a novel technique specifically designed to image collagen. This method allows noninvasive quantification of fibrotic burden in mouse, pig, and human kidneys with high accuracy and speed, and has been successfully demonstrated in settings mimicking human kidney transplantation, suggesting strong potential for clinical translation in donor kidney evaluation [123, 124]. Regarding vascular permeability (VP) assessment, a dual‐wavelength PAM approach combined with spectral unmixing has been developed to overcome the limitations of single‐wavelength methods. This technique accurately distinguishes hemoglobin and Evans Blue absorption signatures, enabling precise VP dynamics assessment. Studies in murine fibrosis models utilizing this method have demonstrated that fibrosis leads to reduced vessel density and increased vessel diameter (Figure 4G), providing valuable insights into microvascular changes during disease progression [125].

In summary, PAM provides a versatile platform for kidney disease research, enabling quantitative assessment of microvascular function, visualization of pathological deposition processes, and evaluation of tissue metabolism. Future developments involving more sensitive probes, faster imaging systems, and integration with multimodal datasets will further solidify PAM's role in advancing nephrological research and clinical practice.

### 4.3 PAM Applications in Tumor Imaging

**FIGURE 5:**
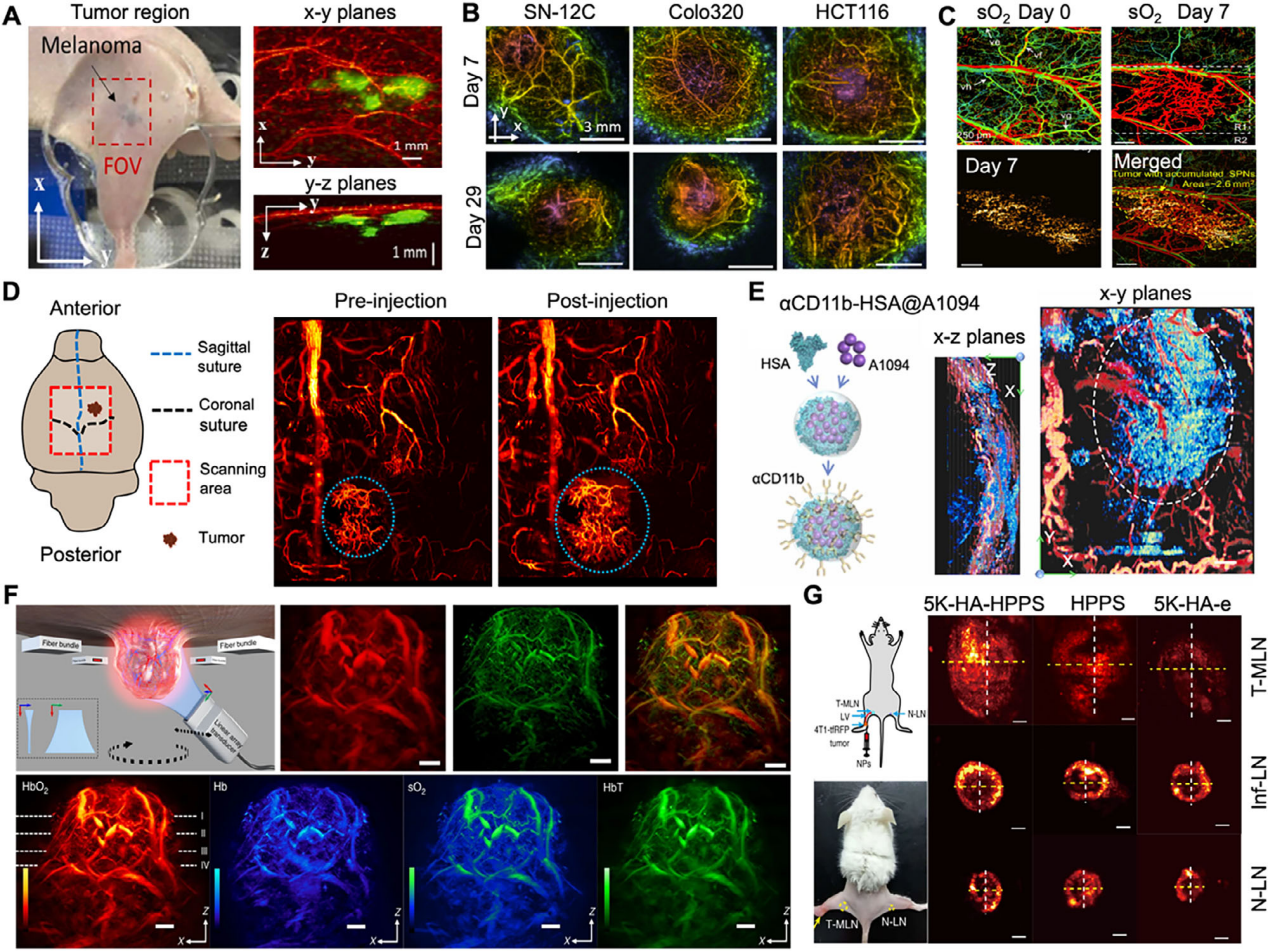
Multimodal PAM for tumor imaging and microenvironment analysis. (A) Photograph and corresponding in vivo OR and AR PAM images of a subcutaneously implanted B16 melanoma in a nude mouse, acquired using a multiscale PAM system. Reproduced under terms of the CC‐BY license [129]. Copyright 2022, The Authors, published by Elsevier GmbH. (B) Vascular PAM images of SN‐12C, Colo320, and HCT116 tumor xenografts captured at selected time points during tumor progression. Reproduced under terms of the Optica Open Access Publishing Agreement from Ref. [130]. Copyright 2022, Optica Publishing Group. (C) Composite image displaying sO_2_ and 1064 nm PAM signals from mouse ear vasculature before cancer cell inoculation and on day 7 post‐injection. Reproduced with permission [131]. Copyright 2025, The Authors, published by Chinese Laser Press. (D) Schematic representation of tumor localization and PAM visualization of cerebral vasculature in a GBM‐bearing mouse before and after intravenous injection of IVTPO. Reproduced with permission [86]. Copyright 2025, The Authors, published by American Association for the Advancement of Science. (E) Schematic of the αCD11b‐HSA@A1094 nanoprobe targeting TAMCs, with PAM images showing TAMCs and adjacent cortical vasculature at 12 h post‐injection on day 18 of tumor development. Reproduced with permission [132]. Copyright 2023, The Authors, published by Elsevier GmbH. (F) MSOM system for in vivo whole‐tumor imaging, with maximum intensity projections of HbO_2_, Hb, THb, and sO_2_. Reproduced under terms of the CC‐BY 4.0 license [82]. Copyright 2020, The Authors, published by Springer Nature. (G) Strategy for LN metastasis detection using intratumorally injected nanoparticles, and PAM images of 5K‐HA‐HPPS distribution in LNs under different pathological states. Reproduced under terms of the CC‐BY 4.0 license [135]. Copyright 2020, The Authors, published by Springer Nature.

PAM has emerged as a transformative tool in oncology, enabling high‐resolution visualization of tumor morphology, functional dynamics, and molecular biomarkers [126]. By mapping optical absorption contrasts at depth, PAM provides unique insights into tumor vasculature, metabolism, and specific molecular events, supporting applications from early detection to therapy monitoring [127, 128]. A high‐speed, wide‐field multiscale PAM system incorporating a custom polygon scanner achieved a more than 12‐fold increase in imaging speed, enabling visualization of subcutaneous melanoma vasculature and tumor morphology at depths up to 1.6 mm (Figure 5A) [129]. Beyond structural imaging, PAM enables functional phenotyping of tumors, as demonstrated in xenograft models (SN‐12C, HCT‐116, Colo320), where it revealed distinct heterogeneities in vascular architecture and oxygenation status (Figure 5B) [130]. Multi‐contrast PAM systems further integrate imaging of microvasculature, oxygen saturation, and targeted nanoprobes to identify hallmark signs of tumorigenesis—such as angiogenesis, metabolic abnormalities, and probe accumulation (Figure 5C)—offering a comprehensive tool for early detection and staging [131].

Deep‐tissue imaging has been a major challenge in optical microscopy. A study showed that PAM can achieve high‐resolution imaging of gliomas in mice through an intact skull using a tumor‐targeting probe, allowing accurate prediction of tumor growth (Figure 5D). The system identified tumor locations and detailed capillary structures, combining deep penetration with high resolution for non‐invasive intracranial tumor imaging [86]. Moreover, PAM serves as a valuable tool for the longitudinal monitoring of local immune activity in tumors. Utilizing an αCD11b protein probe in the second near‐infrared window, it provides exceptional contrast for visualizing tumor‐associated myeloid cell infiltration alongside microvascular and BBB dynamics (Figure 5E) [132]. Tumor spatial heterogeneity is effectively characterized by PAM. A photoacoustic mesoscopy study of breast cancer uncovered distinct regional patterns in oxygenation, vascular structure, and perfusion (Figure 5F), providing valuable insights into intratumorally variability [82]. At the molecular level, PAM visualizes key biomarkers with high specificity. A radiometric photoacoustic probe (BXOS4) enabled micromolar‐resolution imaging of peroxynitrite (ONOO^−^) in tumors, revealing its distribution and supporting studies of cancer progression [133]. Similarly, a near‐infrared radiometric nanoprobe achieved quantitative detection of gamma‐glutamyl transpeptidase (GGT) activity in real time, with a detection limit of 0.48 U/mL, facilitating non‐invasive enzyme activity assessment in vivo [134].

In surgical contexts, PAM aids in accurate sentinel lymph node (SLN) identification. A dual‐targeting nanoparticle (5K‐HA‐HPPS) allowed rapid SLN accumulation and, via PAM, revealed differential distribution patterns between metastatic, normal, and inflamed lymph nodes (Figure 5G) [135]. For early tumor detection, OR‐PAM identified vascular changes in incipient 4T1 breast tumors, including elevated blood oxygen saturation, increased flow velocity, and morphological alterations such as vascular dilation and tortuosity [136]. PAM's translational potential is increasingly evident in preoperative planning and intraoperative guidance. In breast cancer, integrating photoacoustic/ultrasound imaging with a deep learning radiomics model enabled non‐invasive prediction of molecular subtypes with high accuracy [137]. For intraoperative pathology, a next‐generation PAM microscope generated diagnostic images of fresh tissue within five minutes, showing strong concordance with conventional H&E staining [29, 138]. PAM also supports image‐guided therapeutic strategies. A biomimetic platinum‐based nanoplatform (Pt‐IEICO‐4F@dOMV) permitted PAM‐guided multimodal therapy for glioblastoma, crossing the BBB to enable synergistic photothermal, photodynamic, and chemo dynamic treatments [139]. Furthermore, an all‐in‐one theranostic nanoprobe enabled localized drug delivery and real‐time immune monitoring via PAM, visualizing regulatory T cell dynamics during chemo‐immunotherapy and offering a powerful approach to assess treatment response in situ [140].

In summary, PAM provides a versatile and powerful platform for tumor imaging, spanning molecular detection, functional phenotyping, and clinical translation. Continued integration with artificial intelligence, radiomics, and targeted nanotechnologies will further solidify its role in advancing cancer research and precision oncology.

## 5 Probing Central Nervous System (CNS) Architecture and Disease Progression via High‐Resolution PAM

### 5.1 High‐Resolution Mapping of Brain Structure and Function

The brain is one of the most complex and least fully understood organs, with advancements in neuroscience critically relying on technologies capable of capturing anatomical, functional, metabolic, and molecular information across multiple spatiotemporal scales [141]. While established modalities such as MRI and positron emission tomography provide valuable macroscopic perspectives, PAM offers complementary strengths—including superior spatiotemporal resolution, portability, and the capacity for multi‐scale observation [142, 143]. Its unique capabilities make PAM particularly suitable for neuroscience research, enabling multi‐scale visualization of brain architecture and function through the combination of OR‐PAM and AR‐PAM techniques, which facilitates comprehensive observation from microvascular networks to larger tissue structures [144, 145]. Functional PAM can monitor cerebral responses to various stimuli, tracking hemodynamic and oxygenation changes, while artificial intelligence‐enhanced processing methods further improve image quality. Thus, PAM has made substantial contributions to high‐resolution mapping of brain structure and function [146]. Recent progress in contemporary biomedical imaging has dramatically accelerated our understanding of the brain over the past few decades [147], reflecting neuroscience's ongoing integration of innovative methodologies that provide increasingly informative [132], higher‐resolution [10], faster [148], deeper [149], and multifunctional [150] observations. This evolution has established brain research as a primary application area for newly developed imaging technologies. Capitalizing on its high spatial resolution, Hu et al. implemented a dual‐wavelength PAM approach to quantitatively assess oxygenation levels in cerebral microvasculature through intact mouse skulls [151]. This technique achieves approximately 3 µm lateral resolution, enabling imaging of cortical capillary‐level oxygenation, though with a penetration depth limited to under 1 mm. To optimize image quality while addressing depth constraints, researchers often perform imaging with careful scalp removal while preserving skull integrity.

**FIGURE 6:**
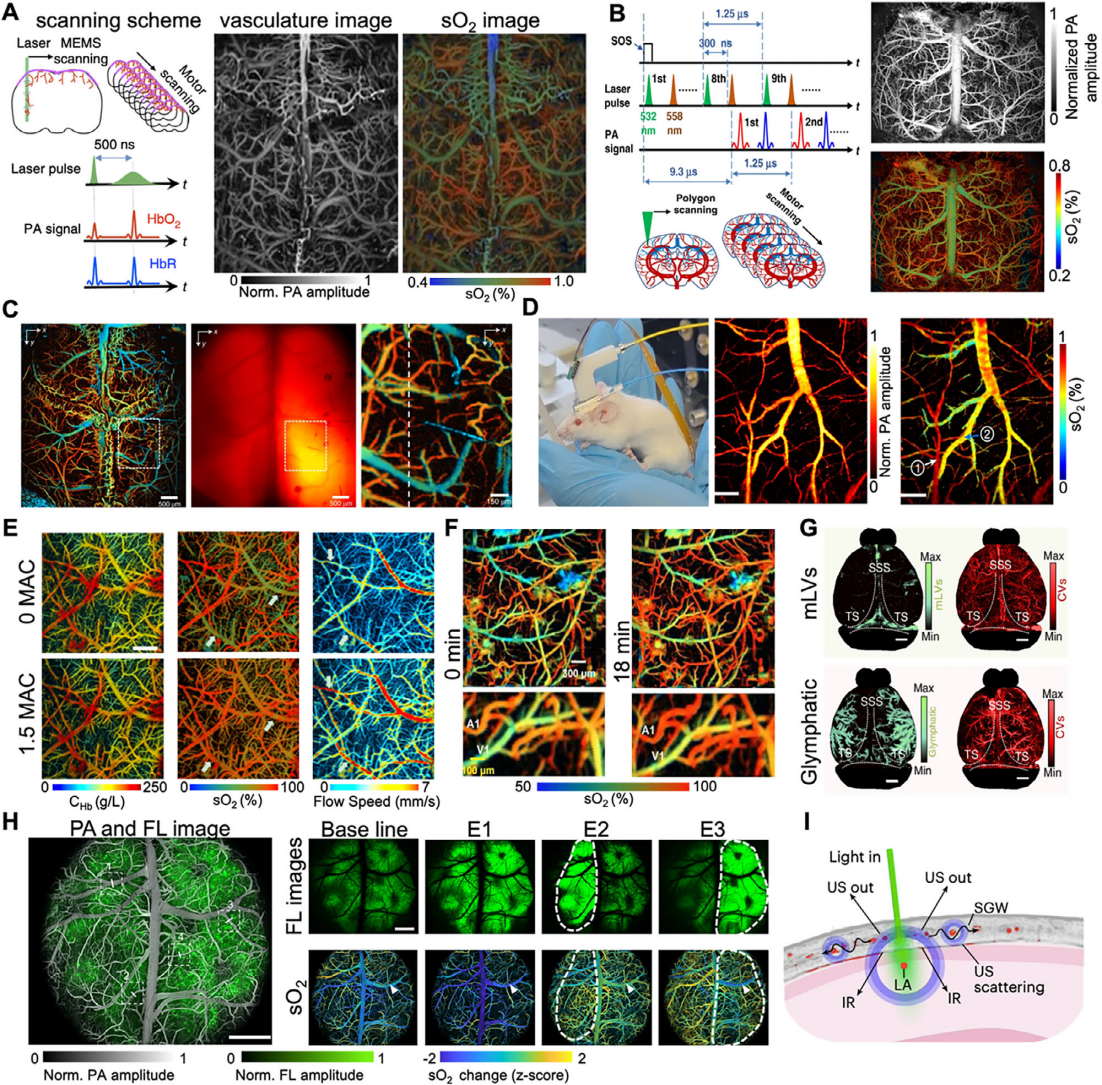
Structural and functional PAM imaging of the central nervous system. (A) Schematic of single‐wavelength pulse‐width‐based functional PAM for rapid mapping of cerebral sO_2_. Reproduced with permission [152]. Copyright 2015, Springer Nature. (B) System configuration and representative images of UFF‐PAM for dynamic monitoring of whole‐brain sO_2_. Reproduced under terms of the CC‐BY 4.0 license [40]. Copyright 2022, The Authors, published by Springer Nature. (C) In vivo visualization of near‐infrared genetically encoded calcium indicators using integrated photoacoustic‐fluorescence microscopy. Reproduced with permission [153]. Copyright 2020, Springer Nature. (D) Freely behaving mouse equipped with a miniaturized PAM probe, with corresponding images showing relative Hb and sO_2_ dynamics in a selected cortical region. Reproduced under terms of the CC‐BY 4.0 license [154]. Copyright 2024, The Authors, published by Springer Nature. (E) Cortical hemodynamics in head‐restrained mice under varying isoflurane anesthesia levels (0 MAC vs. 1.5 MAC), showing changes in C_Hb_, sO_2_, and blood flow velocity. Reproduced with permission [155]. Copyright 2017, Elsevier. (F) PAM visualization of cortical vascular architecture and hemoglobin oxygenation changes in response to systemic epinephrine administration. Reproduced with permission [156]. Copyright 2023, Wolters Kluwer Health, Inc. (G) Co‐registered PAM imaging of meningeal lymphatic vessels (mLVs), glymphatic pathways, and cerebral vasculature (CVs). Reproduced under terms of the CC‐BY 4.0 license [162]. Copyright 2024, The Authors, published by Springer Nature. (H) Spatiotemporal PAM imaging of neurovascular dynamics during epileptic seizures. Reproduced with permission [168]. Copyright 2025, The Authors, published by American Association for the Advancement of Science. (I) Anatomical schematic of transcranial photoacoustic wave propagation through the murine skull. Reproduced with permission [170]. Copyright 2025, Springer Nature.

In this context, PAM has emerged as a particularly potent tool for functional brain imaging, particularly in the mapping of cerebral hemodynamics and sO_2_. To achieve this objective, two distinct methodological strategies have been developed. The first strategy utilizes a single‐wavelength approach that exploits the differential absorption response of hemoglobin to picosecond versus nanosecond laser pulse widths for the quantification of sO_2_ (Figure 6A) [152]. This method obviates the necessity for multi‐wavelength scanning, thereby reducing wavelength‐dependent optical attenuation and significantly enhancing imaging speed and penetration depth. Conversely, a complementary strategy involves the use of ultrafast functional PAM integrated with stimulated Raman scattering to produce dual‐wavelength excitation at 532 and 558 nm. Enhanced by advanced image registration and deep learning algorithms, this configuration facilitates precise quantification of hemoglobin species and high‐resolution visualization of whole‐brain oxygen delivery dynamics, as depicted in Figure 6B [40]. Beyond hemodynamic mapping, functional imaging capabilities have been further extended through the development of innovative near‐infrared genetically encoded calcium indicators (iGECIs) [153]. Their integration with photoacoustic and fluorescence microscopy allows real‐time, high‐resolution monitoring of neuronal activity and concurrent hemodynamics through the intact skull (Figure 6C), achieving lateral and axial resolutions of approximately 3 and 25–50 µm, respectively. This multimodal approach significantly expands the spatiotemporal dimensions accessible for interrogating brain function in vivo.

A critical advancement in the development of PAM is the shift toward miniaturized and wearable systems, which facilitate brain imaging in awake, freely behaving animals and advance the potential for clinical application. For example, Zhong et al. engineered a head‐mounted PAM device weighing merely 4.5 grams, capable of achieving a spatial resolution of 9 µm at a frame rate of 0.2 Hz (Figure 6D). This innovation permits continuous monitoring of cerebral oxygenation and hemodynamics at single‐vessel resolution in freely moving mice [154]. In a related investigation, Cao et al. employed a head‐restrained PAM system in awake mice to conduct functional imaging and metabolic analysis at the microvascular level (Figure 6E), revealing that light anesthesia can partially alleviate hypoxia‐induced reductions in cerebral oxygen metabolism [155]. The selection of anesthetic and vasoactive agents plays a pivotal role in influencing cerebral hemodynamics, a parameter that PAM is particularly adept at monitoring with high precision. For example, a study examining the impact of intravenous epinephrine on the mouse brain utilized functional PAM to uncover a paradoxical response (Figure 6F): the drug caused pronounced constriction of cerebral microvessels and a marked reduction in intravascular oxyhemoglobin, yet simultaneously led to an increase in brain tissue oxygen tension [156]. This observation, likely due to decreased transit time heterogeneity, highlights a complex dissociation between vascular tone and tissue oxygenation, emphasizing the essential role of PAM in elucidating the intricate effects of pharmacological interventions on the cerebral microenvironment. These compact imaging platforms not only minimize motion artifacts and enhance physiological relevance but also create new opportunities for long‐term, behaviorally integrated neural observation. This underscores PAM's evolving role as a versatile and translatable tool in neuroscience research.

### 5.2 Elucidating Brain Pathophysiology and System‐Level Mechanisms

PAM demonstrates growing translational potential by providing critical insights into cerebral pathophysiology under diverse pathological conditions [157]. To meet the need for detailed monitoring of cerebral hemodynamics, a high‐efficiency PAM (HePAM) system was created using a three‐axis optical scanner [158]. It offers an ultrafast B‐scan rate of up to 1.6 kHz, allowing versatile imaging of hemodynamic responses in various brain regions. A key area of application lies in monitoring metabolic disturbances. For instance, a dual‐wavelength PAM technique was developed to rapidly evaluate the effects of metabolic acidosis on the murine brain, quantifying severity‐dependent increases in cerebral microvascular density, diameter, and oxygen metabolism [159]. The study further established a highly accurate diagnostic nomogram, which was also validated in a diabetic mouse model, underscoring PAM's potential for bedside monitoring of acid‐base imbalances and guiding timely clinical interventions. Beyond acute metabolic challenges, PAM has also illuminated the brain's response to physiological stressors. In awake mice subjected to hypercapnia (elevated CO_2_), multi‐parametric PAM revealed vessel type‐dependent increases in diameter and blood flow, accompanied by a decreased oxygen extraction fraction and an overall reduction in cerebral oxygen metabolism [160]. These quantitative findings provide dynamic insights into how the severity and duration of hypercapnia modulate hemodynamic and metabolic responses, helping to resolve long‐standing controversies in the field. A particularly impactful application of PAM is in visualizing and understanding the brain's clearance systems. The recent discovery of meningeal lymphatic vessels has revolutionized the understanding of CNS immune privilege and waste clearance [161]. These vessels facilitate the drainage of macromolecules and metabolic waste from the CNS to the peripheral system via connections to cervical lymph nodes [161]. High‐resolution PAM has enabled whole‐brain in vivo visualization of this system. Using stereoscopic wide‐field PAM, researchers have achieved large‐area imaging of the brain lymphatic system alongside cortical vasculature, clarifying 3D morphological and dynamic details of meningeal lymphatic vessels. Furthermore, dual‐contrast functional PAM has been employed for co‐localized imaging of meningeal lymphatic vessels and cerebral vasculature, distinguishing meningeal drainage from parenchymal glymphatic pathways, as shown in Figure 6G [162]. Building on the theme of AD research, homogeneous‐resolution arched‐scanning PAM has been developed to achieve ultrawide (12 × 12 mm^2^) imaging of the entire mouse cortex, enabling the quantification of early AD‐associated vascular alterations such as increased tortuosity and branch index [163]. These approaches revealed reduced lymphatic drainage volume in Alzheimer's disease mouse models, providing powerful tools for investigating brain lymphatic disorders and advancing the understanding of CNS pathophysiology.

At the core of brain function lies neurovascular coupling (NVC), a fundamental mechanism that dynamically adjusts cerebral blood flow to meet neuronal metabolic demands [164]. This process involves complex interactions among neurons, glial cells, and vascular endothelial cells during neural activation [165]. Conventional techniques, limited by small fields of view or insufficient spatiotemporal resolution, have impeded real‐time, high‐precision observation of NVC across the whole brain [166]. PAM has emerged as a powerful solution to this challenge. Technological innovations in miniaturized PAM systems have been crucial for probing NVC in behaving animals. A dual‐modal imaging probe integrating confocal fluorescence microscopy and PAM—weighing only 1.7 grams—enables high spatiotemporal resolution synchronous neurovascular imaging in freely moving mice [167]. This system captures cerebral blood oxygen metabolism alongside simultaneously recorded neuronal calcium activity, providing novel insights into NVC mechanisms. Subsequent optimizations have led to the development of the linear transducer array‐based hybrid microscope, which combines photoacoustic and confocal fluorescence microscopy to enable cortex‐wide neurovascular imaging in awake mice with subcellular resolution across a 6 × 5 mm field of view, as shown in Figure 6H [168]. This system permits simultaneous monitoring of thousands of neuronal somata and vascular branches, revealing spatiotemporal correlations and functional connectivity in NVC under various conditions. Furthermore, a wearable dual‐modal platform combining PAM and EEG allows simultaneous monitoring of capillary hemodynamics and neural activity in freely moving rats [169]. During seizures, it was found that neurovascular coupling is more strongly correlated under anesthesia than when awake. By enabling precise interpretation of brain functional activity through NVC mechanisms, PAM provides a scientific foundation for developing novel therapeutic strategies for neurological disorders such as Alzheimer's disease and stroke.

In conclusion, the body of work presented in this study highlights that high‐resolution PAM has evolved into an essential tool for investigating the central nervous system. It has significantly enhanced our ability to map brain architecture with microvascular precision, monitor functional dynamics such as hemodynamics and oxygenation, elucidate pathophysiological processes in conditions ranging from metabolic disorders to Alzheimer's disease, and unravel system‐level mechanisms including neurovascular coupling and meningeal lymphatic clearance [199]. Despite the promising trajectory toward clinical translation, the challenge posed by the skull bone remains a substantial barrier to transcranial applications [170]. The path to clinical application faces the challenge of the skull, a major barrier to transcranial light and sound imaging (Figure 6I). Despite advances in super‐resolution and hybrid optoacoustic techniques, their effectiveness is hindered by limited knowledge of the skull's acoustic properties. Progress in this field depends on developing models and strategies to address skull distortions, enabling reliable non‐invasive brain imaging.

## 6 PAM Applications in Whole‐Body Small Animal Imaging and Clinical Translation

Advancing from microscopic cellular observations to a comprehensive systemic understanding represents a pivotal achievement in biomedical research. This section is divided into two parts to outline the translational pathway of PAM. The first part focuses on the role of PAM in preclinical research, emphasizing its application in high‐resolution whole‐body functional and molecular imaging in small animal models, which is essential for validating disease mechanisms and evaluating therapeutic efficacy. The second part addresses the emerging frontier of human clinical application, discussing its use in dermatology, oncology, vascular medicine, and intraoperative guidance, while also examining the ongoing challenges that hinder widespread clinical adoption. The first clinical prototype of PAI was introduced in 2001 for in vivo breast cancer screening, marking a significant milestone in clinical diagnostics [171]. Over the following two decades, human applications of PAI have made substantial progress, creating new opportunities for early disease detection and precision therapy [172]. In clinical settings, photoacoustic computed tomography is more commonly employed due to its superior penetration depth [173], while PAM, with its relatively limited penetration, finds primary application in dermatology and superficial tissue imaging.

### 6.1 Whole‐Body Functional and Molecular Imaging in Small Animals

**FIGURE 7:**
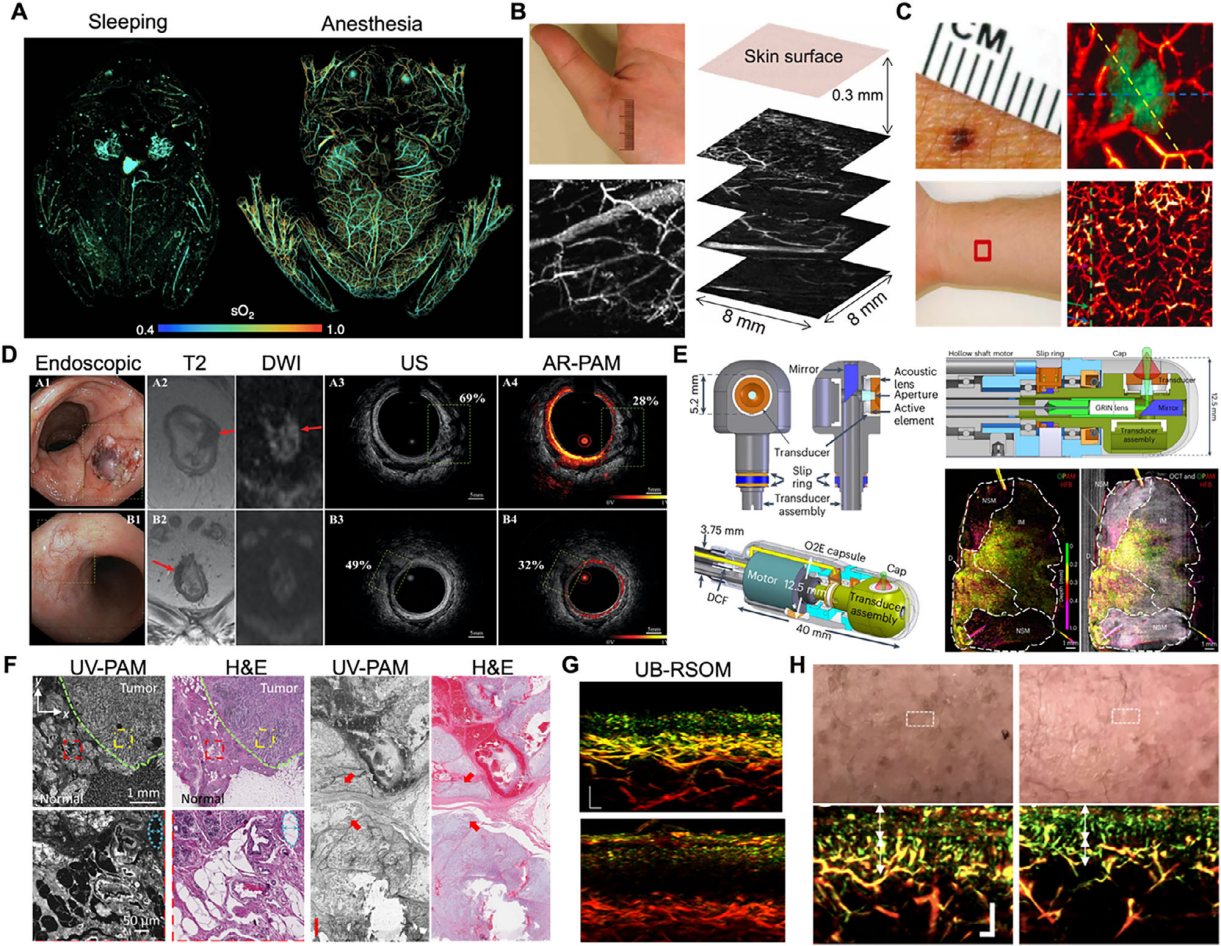
Whole‐body small animal imaging and clinical translation of PAM. (A) Comparative PAM imaging of red blood cell (RBC) perfusion within the same frog's vasculature during sleep and under anesthetic conditions. Reproduced with permission [176]. Copyright 2022, The Authors, published by American Association for the Advancement of Science. () In vivo fPAM at 584 nm wavelength visualizing total hemoglobin concentration in subcutaneous vasculature of the human palm. Reproduced with permission [10]. Copyright 2006, Springer Nature. (C) Photograph and corresponding PAM images acquired from a volunteer's palm using 584 nm laser excitation. Reproduced with permission [177]. Copyright 2025, The Authors, published by Society of Photo‐Optical Instrumentation Engineers. (D) Multi‐modal comparison of endoscopic, T2‐weighted MRI, diffusion‐weighted MRI, ultrasound, and co‐registered PAM/US images from tumor beds in human rectal walls. Reproduced with permission [183]. Copyright 2025, The Authors, published by the Radiological Society of North America. (E) Tethered O2E capsule and its application in human oral mucosa imaging. Reproduced under terms of the CC‐BY 4.0 license [186]. Copyright 2025, The Authors, published by Springer Nature. (F) Comparative analysis between UV‐PAM and H&E staining of thin tissue sections. Reproduced with permissions [29, 138]. Copyright 2017, The Authors, published by American Association for the Advancement of Science; Copyright 2022, Springer Nature. (G) Cross‐sectional ultra‐broadband raster‐scanning UB‐RSOM images from two distinct psoriatic patients. Reproduced with permission [189]. Copyright 2017, Springer Nature. (H) Clinical and second‐generation UB‐RSOM imaging of psoriatic skin in a patient undergoing biologic therapy. Reproduced with permission [190]. Copyright 2022, American Association for the Advancement of Science.

Prior to clinical translation, PAM has established itself as an indispensable tool for non‐invasive, high‐resolution whole‐body imaging in small animal models, providing critical insights into disease mechanisms and therapeutic efficacy. To address the limitations of conventional AR‐PAM in achieving wide‐field imaging, particularly due to its constrained field‐of‐view and limited imaging speed, researchers have integrated a one‐axis waterproof MEMS scanner with a bilinear stepper motor platform [174]. This integration facilitates super‐wide field scanning. The enhanced AR‐PAM system successfully achieves a field‐of‐view measuring 36 × 80 mm^2^, offering potential for high‐resolution imaging of superficial tissues in both small animal models and human subjects. Its utility has been extended to the field of entomology, where PAM has been employed for the non‐invasive morphological analysis of insects, such as ants [175]. This technique addresses the limitations of traditional methods, which often result in sample damage and provide only superficial information. PAM facilitates the detailed examination of both external structures (e.g., antennae, compound eyes, and mandibles) and internal organs (e.g., social stomach, hindgut) through depth‐resolved cross‐sectional imaging, thereby underscoring its significance for comprehensive morphological investigations. Notably, Taboada et al. have advanced high‐resolution PAM methodologies to effectively monitor red blood cell distribution in vivo within glassfrogs (Figure 7A), enabling the quantification of variations in oxygenated and deoxygenated hemoglobin concentrations within the circulatory system [176]. This study illustrates PAM's capability to provide deep, non‐invasive, and quantitative hemodynamic data throughout the entire organism, thereby broadening its applicability across diverse biomedical research contexts.

### 6.2 Clinical Applications in Dermatology, Oncology, and Vascular Medicine

The clinical application of PAM in human microvascular imaging began with a pivotal 2006 study demonstrating high‐resolution in vivo imaging of subcutaneous vessels in the human palm [10], establishing safe functional PAM imaging with laser fluence well below ANSI safety limits. The system achieved a vessel‐to‐background ratio of 35 ± 2 and an average contrast‐to‐noise ratio of 51 dB, visualizing vessels from 350 µm down to the system resolution limit of 50 µm as shown in Figure 7B.

In dermatology, PAM enables noninvasive 3D imaging of human skin microvasculature and pigmented lesions (Figure 7C), effectively distinguishing between acral and non‐acral vascular networks while delineating key skin layers. Clinical studies have confirmed its remarkable accuracy in measuring thickness and microvascular characteristics of melanocytic nevi, with results closely correlating with histological findings [177]. Extending beyond diagnostic imaging to therapeutic monitoring, volumetric PAM serves as a powerful tool for quantifying pharmacodynamic responses. For instance, it has been applied to differentially assess the vasoconstrictive effects of various topical corticosteroids across skin depths, providing detailed 3D insights for evaluating pharmaceutical efficacy and bioequivalence directly in vivo [178]. These capabilities highlight PAM's potential for assessing skin microcirculation and pigmented lesions, suggesting promising applications in diagnosing and monitoring systemic microvascular diseases and cutaneous malignancies.

For cardiovascular assessment, PAM shows particular promise in evaluating cutaneous microcirculation. Human volunteer studies have successfully imaged palm microvasculature during induced ischemic challenges, capturing detailed hemodynamic responses including decreased capillary oxygen saturation during occlusion, temporary post‐occlusion hyperoxygenation, and clear hyperemic reactions following ischemia release [179]. Complementing studies on ischemic challenges, PAM offers high‐resolution insights into metabolic vascular dysregulation. It has been utilized to capture transient, selective arteriolar constriction in response to acute hyperglycemia, providing direct in vivo evidence of glucose‐induced endothelial dysfunction and its specific impact on microvascular tone [180]. These observations aligned well with conventional measurement techniques, underscoring PAM's effectiveness for high‐resolution assessment of microvascular function. Further advancing these applications, researchers developed an integrated PAM‐photoplethysmography (PAM‐PPG) system for simultaneous vascular imaging and hemodynamic monitoring [181]. In human finger studies, synchronized PAM images and PPG recordings demonstrated that cardiac‐induced photoacoustic variations corresponded precisely with PPG‐derived heart rates. Complementing these advances, specialized dual‐wavelength OR‐PAM systems have been engineered for human nailfold capillary imaging [182], incorporating technical innovations that significantly enhance sensitivity and practical utility for clinical deployment.

In oncology, PAM demonstrates significant potential for tumor imaging and vascular assessment. Research on rectal cancer detection following chemoradiotherapy has shown particularly promising results through multimodal approaches (Figure 7D). An initial investigation utilized an AR‐PAM/US endorectal probe to train convolutional neural network (CNN) classifiers [183]. The PAM‐based CNN model effectively differentiated residual rectal tumors from normal tissue, achieving an area under the curve (AUC) of 0.98 on previously unseen in vivo patient data, significantly outperforming the US‐based CNN (AUC of 0.71). Building on this foundation, the same research team reported substantial engineering advancements in their co‐registered AR‐PAM/US system [184], achieving critical improvements in resolution, optical coupling efficiency, signal‐to‐noise ratio, and overall system stability. When applied to ex vivo colorectal cancer samples, the enhanced system produced significantly different signals between normal, cancerous, and post‐treatment tissues, strongly corroborating the earlier CNN‐based classifications. While these results are promising, limitations, including constrained penetration depth and system portability require further hardware refinements before full clinical implementation [185]. Recent advancements in PAM are accelerating its clinical use, highlighted by the creation of a dual‐modality O_2_E capsule endoscope for assessing Barrett's esophagus without labels [186]. This device combines a compact, ultra‐broadband endoscopic PAM with OCT for high‐resolution 3D imaging of tissue structures (Figure 7E). Tested on human specimens, the O_2_E platform effectively identified various mucosal types, including dysplasia and intramucosal carcinoma. Its ability to enhance 3D tumor detection in Barrett's mucosa shows significant diagnostic promise, marking a crucial step toward PAM's clinical adoption for human pathology.

### 6.3 Intraoperative and Histopathological Applications

Conventional pathology interpretations relying on histological imaging often delay diagnosis and management due to time‐consuming sample preparation. While imaging cell nuclei in unstained, unprocessed specimens represents an ideal approach, irregular surfaces in fresh tissues present technical challenges. Intraoperative pathology demands vary significantly by tissue type: breast surgeons require rapid microscopic margin assessment to prevent reoperations, while orthopedic oncologists face difficulties obtaining reliable frozen sections of bone, frequently resulting in excessively wide resections based on preoperative imaging. PAM addresses these challenges through multiple innovative approaches. For soft tissue evaluation, PAM provides label‐free, multilayered, histology‐like imaging of lumpectomy surfaces that strongly correlates with hematoxylin and eosin (H&E) staining [138]. This capability enables rapid extraction of diagnostic features including nuclear size and cellular packing density, facilitating immediate margin assessment and reducing re‐excision likelihood (Figure 7F). In bone tissue, UV reflection‐mode PAM permits real‐time 3D contour scanning of thick undecalcified or decalcified specimens without sectioning [29]. Results validated against H&E staining, augmented by generative adversarial network‐based virtual staining, assist pathological interpretation. To enhance intraoperative imaging throughput, parallel UV PAM (PUV‐PAM) was developed for slide‐free specimen evaluation [187]. Utilizing eight simultaneous focused beams, this system achieves rapid imaging at 0.4 mm^2^/sec with 1.3 µm resolution, enabling high‐speed, high‐resolution visualization of fresh tissues even with irregular surfaces. Collectively, these advancements establish PAM as a versatile, slide‐free platform for rapid intraoperative pathology across diverse tissue types [200]. However, for PAM to achieve widespread clinical adoption, challenges such as limited penetration depth, portability, and imaging speed must be addressed through technological advancements. Validation of PAM's efficacy and reliability necessitates larger multicenter trials and standardized protocols. Future research should prioritize developing compact, user‐friendly systems, targeted contrast agents, and AI integration [188].

To complement PAM's superficial imaging capabilities, optoacoustic mesoscopy has emerged as a pivotal technology bridging the gap between microscopic surface imaging and deeper tissue assessment. Operating in the mesoscopic regime, it provides an optimal combination of depth penetration and resolution for visualizing pathophysiological processes within the entire dermis. A key demonstration of this capability utilized RSOM with ultra‐broadband detection [189], enabling label‐free, 3D visualization of vascular patterns and inflammation in psoriasis (Figure 7G). Subsequent refinement of this technology achieved sub‐10‐µm resolution and demonstrated its ability to extract specific 3D inflammatory and morphological biomarkers, quantifying treatment efficacy with sensitivity and accuracy unattainable by conventional clinical scoring as shown in Figure 7H [190]. These advancements position optoacoustic mesoscopy as a complementary modality that effectively addresses the primary clinical limitation of PAM, which is penetration depth. This technique offers quantitative, depth‐resolved biomarkers and enhances the potential for precision medicine in dermatology.

## 7 Conclusion and Perspectives

PAM has firmly established itself as a transformative frontier in biomedical imaging by effectively bridging the critical gap between pure optical microscopy and conventional clinical modalities [191]. Over the past two decades, PAM has evolved from a promising laboratory innovation into a versatile and indispensable platform capable of high‐resolution, multi‐scale interrogation that spans from subcellular organelles to whole‐organ vascular architectures. This evolution has demonstrated a profound impact on both fundamental biological research and emerging clinical applications [192]. This comprehensive review systematically chronicles the synergistic technological advancements propelling PAM forward. Pioneering hardware innovations, which include high‐speed scanning mechanisms, sophisticated optical‐acoustic combiners, multi‐wavelength laser systems, and miniaturized wearable probes, have collectively overcome previous limitations in imaging speed, spatial resolution, and practical deployability [193]. Moreover, strategic multimodal integration with complementary techniques such as OCT, ultrasound, and fluorescence microscopy has facilitated unprecedented correlative visualization of structural, functional, and molecular information within complex biological systems [194]. Concurrently, the strategic incorporation of artificial intelligence and sophisticated computational algorithms has revolutionized image reconstruction and interpretation, enabling real‐time super‐resolution processing, intelligent artifact reduction, and virtual histological staining without the need for physical tissue sectioning [191]. Furthermore, the rational design and development of targeted molecular contrast agents, alongside genetically encoded indicators, have dramatically expanded PAM's molecular profiling capabilities, facilitating mechanistic insights into intricate disease pathways and therapeutic responses at a previously unattainable level of detail [15].

In biomedical research, PAM has fundamentally contributed to elucidating complex physiological and pathological processes. It has decoded neurovascular coupling dynamics and functional brain connectivity with microvascular precision, elucidated spatiotemporal heterogeneity within tumor microenvironments, and quantified subtle vascular dysfunction in metabolic disorders affecting the liver and kidneys [20]. Its unparalleled capacity to achieve subcellular resolution and surpass the optical diffraction limit via advanced super‐resolution techniques underscores its transformative potential for single‐cell analysis and fundamental cell biology [41]. In the clinical realm, PAM demonstrates considerable and growing utility across multiple specialties: in dermatology for non‐invasive vascular and pigmented lesion evaluation; in oncology for tumor angiogenesis mapping and molecular phenotyping; and in intraoperative settings for rapid, label‐free surgical margin assessment [21]. Groundbreaking innovations such as ultrafast, multi‐beam parallel acquisition systems suggest the imminent feasibility of seamlessly integrating PAM into real‐time surgical workflows, potentially reducing reoperation rates and enabling instantaneous diagnostic feedback.

Notwithstanding these remarkable achievements, several formidable challenges must be decisively addressed to facilitate broad clinical adoption and maximize PAM's societal impact. The fundamental resolution‐depth trade‐off persists, particularly constraining high‐resolution imaging in deep‐seated tissues. Imaging speed, despite significant improvements, remains a bottleneck for capturing rapid physiological processes and for large‐volume clinical screening. The current scarcity of clinically approved, target‐specific contrast agents critically restricts the modality's potential for molecular diagnostics and precision medicine applications [195]. Furthermore, practical issues surrounding system portability, cost‐effectiveness, operational complexity, and a lack of standardized imaging protocols and analytical benchmarks continue to impede widespread implementation, especially in resource‐limited settings [196]. In addition to the immediate technical and translational challenges, the field confronts several ambitious grand challenges that will define its future trajectory. A primary scientific goal is to develop real‐time, high‐resolution metabolic imaging at the organelle level. This includes visualizing mitochondrial dynamics to directly elucidate fundamental cellular processes and their dysregulation in disease. From the perspective of clinical adoption, a significant translational challenge lies in establishing a clear regulatory pathway and achieving U.S. Food and Drug Administration approval for PAM‐based clinical endpoints and devices. Such advancements would represent a critical maturation of the technology and would unlock its full potential in routine patient care. Additionally, pioneering efforts are focused on developing integrated “lab‐on‐a‐chip” PAM systems for low‐cost, high‐throughput point‐of‐care diagnostics. Furthermore, the creation of comprehensive digital tissue atlases by correlating multiscale PAM data with other omics datasets aims to provide a holistic view of tissue pathophysiology.

Looking ahead, the future trajectory of PAM hinges on integrated, multidisciplinary advancements converging across several key domains. Deep learning‐based image reconstruction and automated diagnostic algorithms will be pivotal for enabling real‐time, operator‐independent visualization and quantitative biomarker extraction. The development of compact, user‐friendly, and cost‐effective devices—including handheld and wearable systems—will be crucial for accelerating clinical translation beyond specialized academic centers [197]. The advent of safer, highly specific contrast agents, coupled with the strategic development of integrated multi‐modal systems that synergistically combine PAM with MRI, Raman microscopy, and other modalities, will unlock comprehensive, multi‐parametric insights into tissue structure, function, and molecular composition. Large‐scale, rigorously designed multicenter trials are imperative for establishing standardized operational protocols and validating clinical efficacy across diverse patient populations [198].

In terms of application prospects, several domains stand out for their high translational potential: real‐time intraoperative guidance for oncological and neurological surgeries; longitudinal monitoring of treatment response in chronic diseases and cancer therapy; early disease detection through sensitive microvascular and molecular profiling; and personalized risk stratification based on functional imaging biomarkers. As continuous, synergistic innovations unfold across instrumentation, contrast agent chemistry, and computational analytics, PAM is poised to transcend its current status as a niche research tool, evolving into a pivotal component of the precision medicine paradigm. Its seamless integration across the research‐to‐clinical continuum promises to fundamentally improve early diagnosis, therapy personalization, and ultimately, patient outcomes across a diverse spectrum of medical disciplines.

## Conflicts of Interest

The authors declare no conflicts of interest.
